# Reconnecting Cities to the Biosphere: Stewardship of Green Infrastructure and Urban Ecosystem Services

**DOI:** 10.1007/s13280-014-0506-y

**Published:** 2014-04-17

**Authors:** Erik Andersson, Stephan Barthel, Sara Borgström, Johan Colding, Thomas Elmqvist, Carl Folke, Åsa Gren

**Affiliations:** 1 Stockholm Resilience Centre, Stockholm University, 10691 Stockholm, Sweden; 2 The Beijer Institute, Royal Swedish Academy of Sciences, PO Box 50005, 10405 Stockholm, Sweden

**Keywords:** Biodiversity, Ecosystem services, Property rights, Stewardship, Urban ecology, Urban social–ecological systems

## Abstract

Within-city green infrastructure can offer opportunities and new contexts for people to become stewards of ecosystem services. We analyze cities as social–ecological systems, synthesize the literature, and provide examples from more than 15 years of research in the Stockholm urban region, Sweden. The social–ecological approach spans from investigating ecosystem properties to the social frameworks and personal values that drive and shape human interactions with nature. Key findings demonstrate that urban ecosystem services are generated by social–ecological systems and that local stewards are critically important. However, land-use planning and management seldom account for their role in the generation of urban ecosystem services. While the small scale patchwork of land uses in cities stimulates intense interactions across borders much focus is still on individual patches. The results highlight the importance and complexity of stewardship of urban biodiversity and ecosystem services and of the planning and governance of urban green infrastructure.

**Electronic supplementary material:**

The online version of this article (doi:10.1007/s13280-014-0506-y) contains supplementary material, which is available to authorized users.

## Introduction

The rate of urban growth is unprecedented. The Earth System has become urbanized in the sense that decisions by the majority of the human population now living in cities affect the resilience of the entire planet (Seto et al. 2011). Urban demand for ecosystem services is a major driver behind global environmental change but the choices people make are often disconnected from their environmental imprint in distant places (Folke et al. 1997; Grimm et al. 2008). Much of urban growth has been at the expense of the capacity of terrestrial and marine systems to generate and sustain essential ecosystem services (Foley et al. 2005) and is currently challenging biophysical planetary boundaries for the world as we know it (Rockström et al. 2009). There is an urgent need to reconnect people in urban areas to the biosphere (Folke et al. 2011).

Contemporary society, urban lifestyles, and changes, such as the decline of traditional land uses in the peri-urban landscape, have changed the way people in cities perceive and interact with the biosphere (Turner et al. 2004). The physical and mental distance between urban consumers and the ecosystems supporting them mask the ecological implications of choices made (Rees and Wackernagel 1996; Folke et al. 1997). Instead of oblivious consumers, cities need engaged stewards that can help redirect urbanization into a driver of positive change for humanity and the life-supporting systems that we depend upon. But how can people living in cities with urban lifestyles be reconnected to the biosphere? How do we ensure sustainable generation, management, and governance of ecosystem services for human well-being in cities, as well as ensure that cities contribute to incentives for better stewardship of distant landscapes and seascapes?

Though providing but a fraction of the ecosystem services consumed, urban landscapes represent key arenas for learning about the way humans interact with the environment and what sustainable ecosystem stewardship might entail (Miller 2005; Chapin et al. 2010). The focus of this paper is on lessons learnt for stewardship of ecosystem services within urban social–ecological systems (Berkes and Folke 1998). We draw on more than 15 years of empirical work within cities in relation to ecosystem service generation in urban landscapes, particularly regulating ecosystem services, and their stewardship with enabling institutions (e.g., property rights), social networks and involvement of local user groups and civil society in green area management and governance. Our work in the Stockholm urban landscape, Sweden, has helped reveal green areas and ecosystem services not previously perceived in urban planning and clarified mismatches between institutions, governance, and urban ecosystems for human well-being (Electronic Supplementary Material, Appendix S1). We emphasize that an urban social–ecological approach (Electronic Supplementary Material, Appendix S2) reduces the tension between conservation and city expansion and provides directions for shifting urbanization patterns toward sustainability. We recognize that most of our empirical work is from one particular city set in a certain context, but believe that our concluding propositions for urban resilience building can communicate with other cities and inspire theoretical discussions.

## Urban Social–Ecological Systems

### The Urban Landscape

Often, green space in urban areas can be remnants of a cultural landscape with biodiversity-rich habitats (Barthel et al. 2005). Many cities incorporate prime habitats that sometimes are rare in the larger region. For example, in regions where land-use intensification has led to loss of landscape diversity and habitats, such as ponds and non-cultivated elements, cities subjected to other drivers have become refuges for species associated with these habitats (Colding and Folke 2009). However, biodiversity and landscape heterogeneity in cities should not only be seen in relation to surrounding hinterlands. Urban landscapes have evolved under extremely complex influences of changing land uses and management practices, sustaining some habitats and fundamentally altering others. We need a detailed understanding of what “green” infrastructure really means in the urban context as well as how the values have come to be (Kinzig et al. 2005; Colding et al. 2006).

Cities are rife with “novel ecosystems” (Hobbs et al. 2006), which deserve to be acknowledged for the values they possess in terms of biodiversity and ecosystem services. Comprehensive analyses of urban green spaces have shown that land uses such as private and public gardens, cemeteries, old brown-fields, and golf courses may contribute significantly to ecosystem services provided by the urban landscape (Colding et al. 2006; Goddard et al. 2010). Incentives, interests, and ambitions among managers and stakeholders and the institutional framework set the stage for management of such spaces and their ecosystem services (Andersson et al. 2007). Over time, this close interaction between human actors, the social context in which they are embedded and the landscape may lead to biodiversity-rich systems maintained as much by human stewardship (Barthel et al. 2005, 2010), the protection of land by the state (Borgström 2009), civil society, and socioeconomic factors (Hope et al. 2003) as by ecological processes.

Urban landscape mosaics are often characterized by small land-use patches and high heterogeneity. It has been suggested that landscape structure becomes ecologically important only when a certain habitat drops below a threshold level coverage (Andrén 1994). This means that spatial structure becomes a key concern in cities, both as ecological networks and adjoining areas (Colding 2007; Andersson and Bodin 2009). Even if there are calls for more integrated landscape approaches in urban planning (Poiani et al. 2000), those commonly concern the large scale green structure and as a result leave out the potential and small scale patches within the built up areas (Colding et al. 2006). These integrated approaches also have to overcome the organization of urban policy that is characterized by a multitude of separate sectors and that fail to acknowledge the complexity of urban social–ecological systems (Runhaar et al. 2009). Issues relating to urban ecosystem services involve a wide range of actors seldom adding up to a comprehensive whole (Ernstson et al. 2010).

Furthermore, when addressing issues of biodiversity, both urban planning and nature conservation policies tend to focus on the establishment of set-asides using formal protection with strong focus on threatened species and their habitats. Such approaches risk reinforcing the land-use dichotomy of conservation versus exploitation and simply miss and exclude many ecologically important land uses, their ecosystem services and the local stewards engaged (Colding et al. 2006). The location of urban protected areas is often the result of intricate negotiations between ecological, economic, and social interests. In many cases, the politics of decision-making processes makes it more difficult to muster arguments for protection and ecological recognition of such areas that are make sure that sites attractive for urban real-estate developers (Ernstson et al. 2008; Borgström 2009).

### Ecosystem Service in Urban Areas

Green infrastructure in cities generates a diversity of ecosystem services (Jansson and Nohrstedt 2001). While we begin to understand the importance of urban green areas we still have a limited understanding of the mechanisms behind the generation of urban ecosystem services. The most commonly articulated link between urban green space and human well-being in current urban planning is through so called cultural services, e.g., recreation and health (Tzoulas et al. 2007). Also provisioning services, like food production in, for example, home gardens (Altieri et al. 1999; Krasny and Tidball 2009) and links to biodiversity conservation have been in focus (Goddard et al. 2010; van Heezik et al. 2012).

**Fig. 1:**
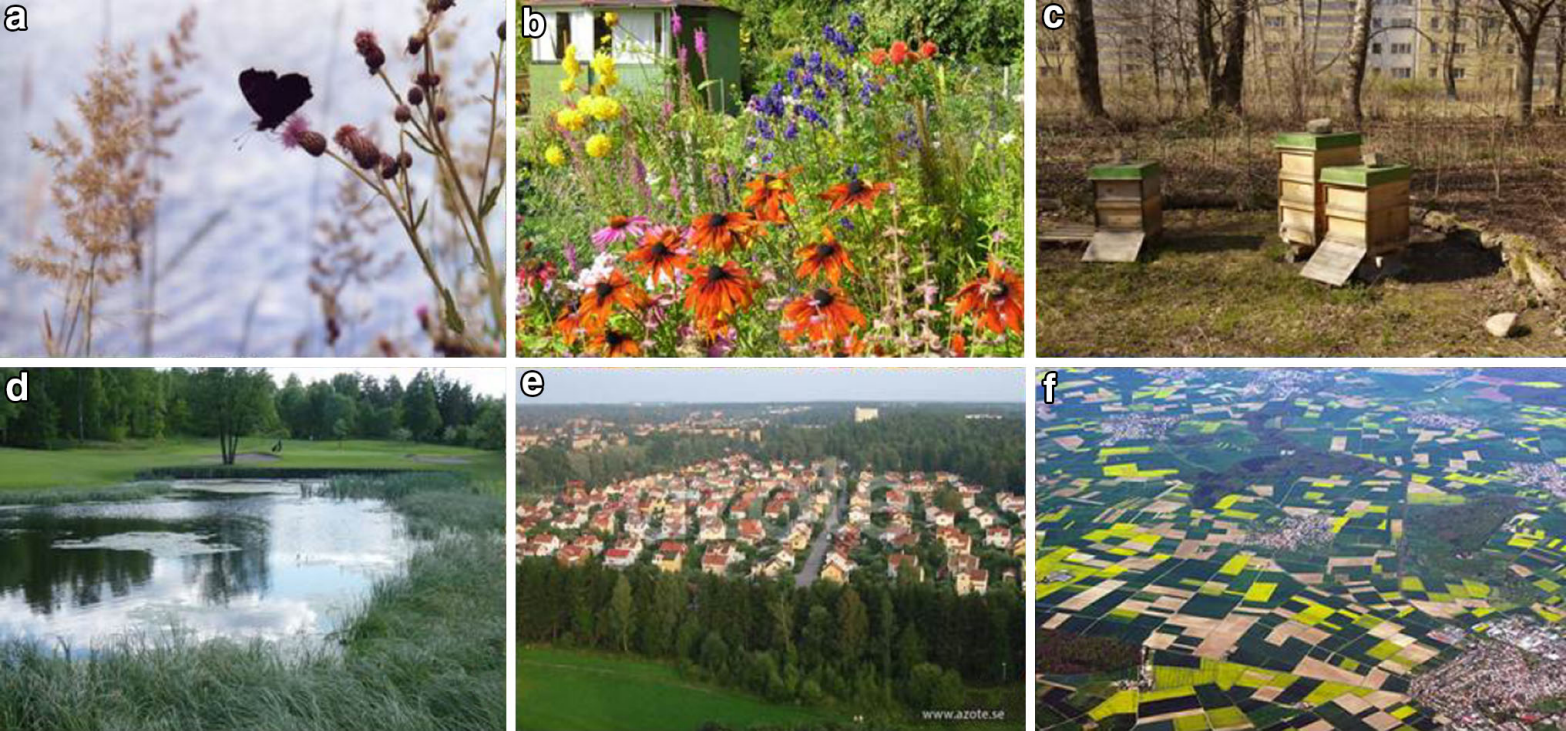
Local user groups and stewardship of regulating ecosystem services in urban green areas. **a** Domestic gardens support biodiversity and species of significance in, e.g., pest control and seed dispersal (photo Carl Folke). **b** Allotment gardens provide critical habitats and food sources during vulnerable animal life history stages (photo Stephan Barthel). **c** Community gardens generate ecosystem services like pollination that spill over into the wider landscape (photo Johan Colding). **d** Urban golf courses function as stepping stones for keystone species with ponds hosting amphibians including endangered and keystone species (photo Stefan Lundberg). (e) Trees improve air quality and sequester carbon (photo Azote). **f** Green spaces within cities consist of remnants of biodiversity-rich cultural habitats in an otherwise fragmented landscape (photo Jakob Lundberg)

The studies reported here focus on the link to human well-being through regulating ecosystem services, such as seed dispersal, pest regulation, and pollination. These services are generated by complex interactions in urban social–ecological systems, and not by ecosystems alone (Andersson et al. 2007) as human activities may both promote service providers (Kremen 2005) and make services available to the beneficiaries (Fig. 1). This serves to illustrate the connection between biodiversity and ecosystem services (Kremen 2005) and the role of biodiversity for social–ecological resilience also in urban areas.

Many ecosystem services need to be locally provided in urban landscapes for easy access and use by a greater set of city-inhabitants, e.g., daily nature encounters, noise reduction, absorption of pollutants in water and air. The small size of many urban land-use patches make it difficult if not impossible to promote the generation of the full range of desired ecosystem services within individual patches. A closer investigation of regulating services reveals spatial and temporal interdependencies seldom recognized by governance structures. Many regulating services, including seed dispersal, pest regulation, and pollination, are not restricted to the areas where they originate but transcend habitat boundaries and affect also the surrounding landscape (Jansson and Polasky 2010; Blitzer et al. 2012). Such services may depend on functional connectivity (Fahrig et al. 2011) between different habitats, implying that a landscape perspective on management and planning for urban ecosystem services is often necessary (Colding 2007; Ernstson et al. 2010).

For example, Lundberg et al. (2008) showed how the preservation of a highly valued recreational oak-dominated landscape benefits from seed dispersing birds that also need coniferous forest. The coniferous forests tend to be located outside the recreational landscape and separated from it by administrative boundaries. Jansson and Polasky (2010) quantified the change in an ecosystem service over time and demonstrated how temporal dynamics may unintentionally erode the capacity to grow alternative crops in an agricultural system. Non-cultivated lands together with rape fields could sustain pollination and pollinator diversity, but were insufficient in themselves to maintain all pollinator species during periods of cereal production. The study showed how failure to address such dynamics eroded social–ecological resilience. By losing some of the pollinator species, the potential for response diversity diminished, making the regulating service more vulnerable to disturbance and change (Elmqvist et al. 2003).

The appreciation of green infrastructures in cities is often manifested in higher house prices close to green areas (Wittemyer et al. 2008). But appreciation and use as they are expressed today raise concerns about the long-term generation of ecosystem services and in particular regulating ecosystem services. For example, when green areas attract adjoining urban development they risk becoming isolated and thereby losing some of the biodiversity and related services that made them attractive in the first place (Borgström et al. 2012). Furthermore, high human population density and limited space in cities often result in demand for multifunctionality of green space, where stewardship of ecosystem services is confronted with multiple objectives, meanings, and conflicting interests (Borgström 2009; Ernstson and Sörlin 2009).

### The Formation of Stewardship of Urban Ecosystem Services

**Fig. 2:**
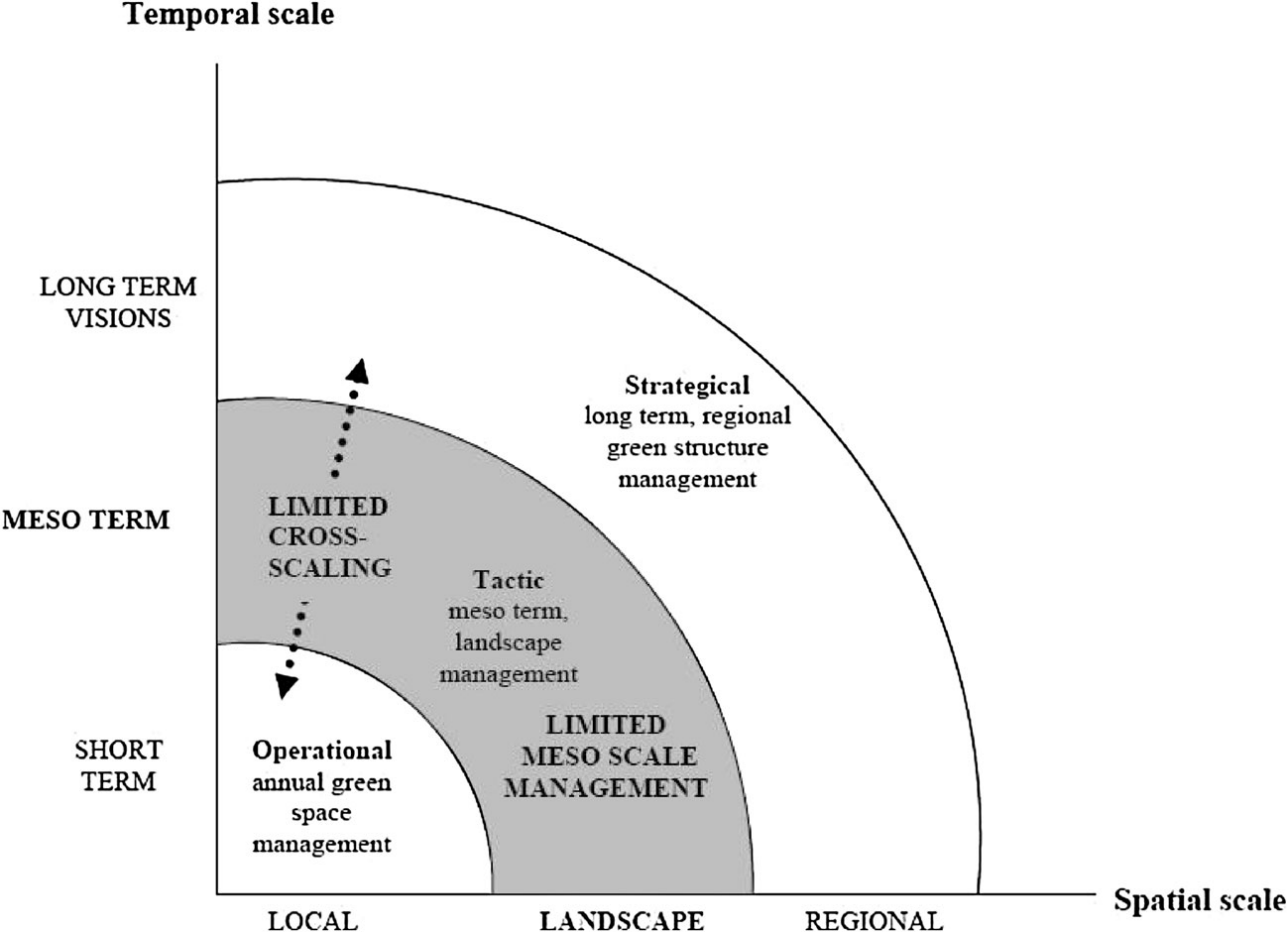
Comparatively little attention is paid to the meso-scale and cross-scale interactions are not recognized among planners and managers of urban green infrastructure (from Borgström et al. 2006)

Increasing people’s awareness of how their actions impact the biosphere is not just a matter of close proximity to green areas, stewardship is about getting involved, which in turn may be facilitated by institutional designs and social movements. Today’s institutions poorly match current changes in urban ecosystems (see Fig. 2; Borgström et al. 2006). Prospects for governance of urban ecosystem services, which strongly benefit from local stakeholder involvement, are becoming further limited when property rights systems change due to urbanization. Property right dynamics shaping human relationships to land can be quite influential, e.g., by helping counteract the growing disconnection of urban residents from nature (Pyle 1978). However, property right arrangements for the green infrastructure that produce urban ecosystem services seldom receive attention in urban settings in competition with other land uses. The global trend of privatization of public land in cities (Lee and Webster 2006) restricts people’s ability to practically engage with urban ecosystems and their services, and if associated with loss of diversity this development might constrain the capacity to deal with change in effective ways. Common property systems, by which groups or a community of resource users share a common interest in resource management (Ostrom 1990), are rare in relation to urban ecosystems. This further reduces the opportunity for people and groups in cities to have meaningful interaction and provide stewardship of their local ecosystems (Andersson et al. 2007; Colding and Barthel 2013).

Diverse and new forms of property rights arrangements hold potential to play a much greater role in stewardship of urban landscapes than has hitherto been recognized (Colding and Barthel 2013). Furthermore, institutional diversity may not only increase diversity of land management approaches (Andersson et al. 2007), but also enhance self-organization of urban systems to adaptively deal with change, i.e., their social–ecological resilience (Folke et al. 2003). As institutional research suggests, having a multitude of property rights regimes that fit the cultural, economic, and geographic context in which they are to function (Hanna et al. 1996) appears also to be critical for resilience building of cities (Colding and Barthel 2013).

An important motivation for civic groups, at least the more affluent, to engage in stewardship of ecosystems in urban landscapes is sense-of-place, memory, and meaning (Andersson et al. 2007; Barthel et al. 2010). Social–ecological memory encapsulates the means by which knowledge, experience, and practice of ecosystem stewardship are captured, stored, revived, and transmitted through time (Barthel et al. 2010). For instance, in collectively managed gardens, community engagement results in a shared history manifested in artifacts, locally adapted organisms, trees, landscape features, and written accounts (Nazarea 2006; Barthel et al. 2010). These objects tend to outlive the practices that first shaped them and function as shared memory carriers between people and across generations (Barthel et al. 2010). Different forms of participation also carry shared memories, such as exchange of seeds for planting and oral traditions, which in combination with physical objects guide a portfolio of practices for how to deal with a changing social–ecological context, and local responses to such fluctuations. For instance, in some garden communities, a small percentage of 1 year’s harvest is often saved for the next planting. Over time, this enhances the probability of locally adapted varieties of crops co-evolved with human practices and local environmental conditions. Social–ecological memory in collectively managed gardens, for example, is favorable for the conservation of ecosystem service providers (Kremen 2005) normally associated with rural landscapes.

**Fig. 3:**
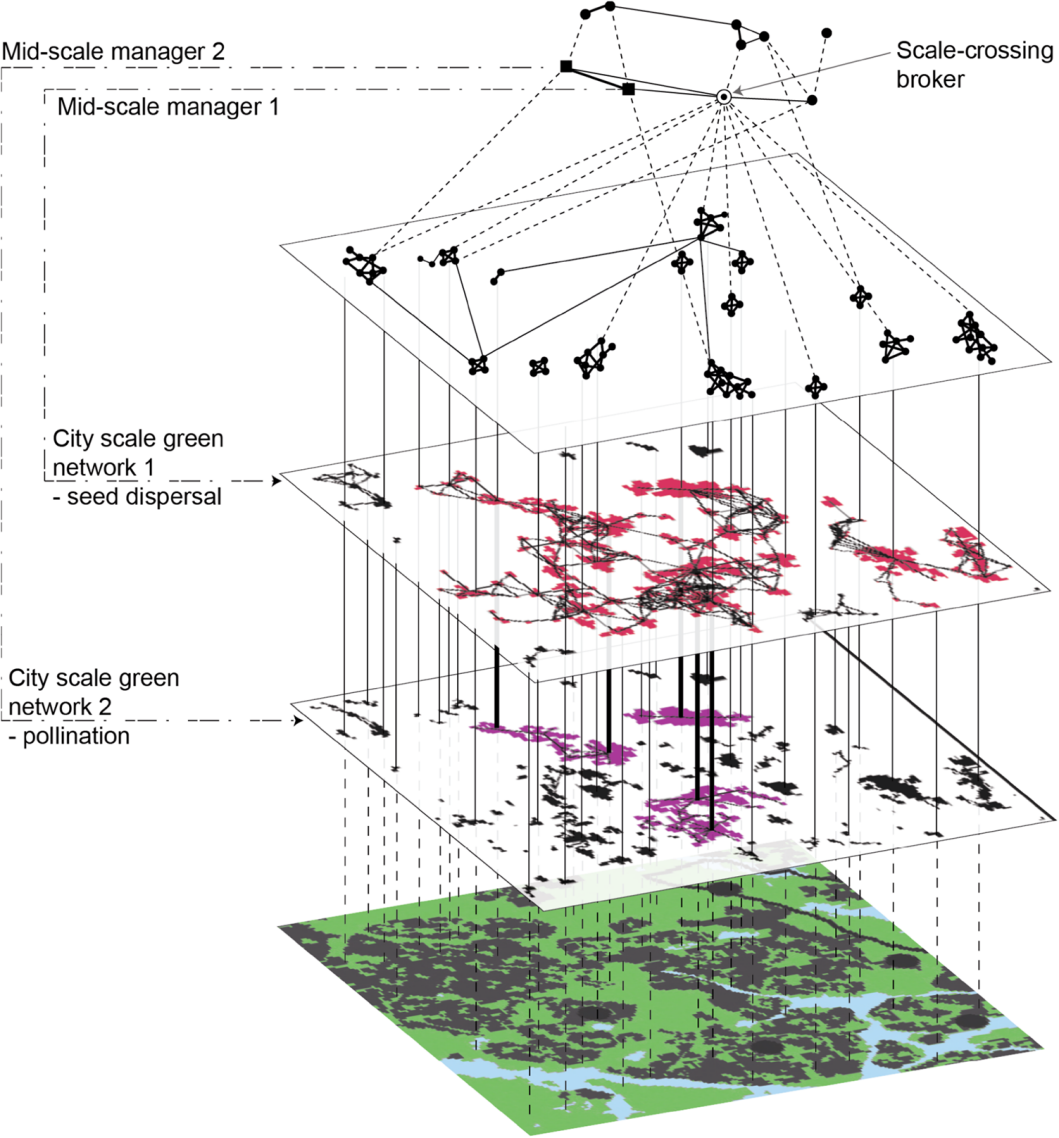
To support the continuous generation of urban ecosystem services, governance structures are needed that connect local experiential knowledge of ecosystem management with those of higher scale understanding outlined in the figure. In such arrangements, the broker position in social networks should be identified and strengthened since it may be needed to link ecosystem stewards across scales, and on different sides of sectoral and administrative boarders. Such scale-crossing brokers might be complemented with more ecologically focused mid-scale managers (Ernstson et al. 2010) (figure from Ernstson et al. 2010)

Current urban green spaces tend primarily to be managed at the local scale, where within-site qualitative characteristics are the focus (Borgström et al. 2006; Andersson et al. 2007; Ernstson et al. 2010). However, the spatial and temporal dynamics of ecosystem services often demand co-operation and co-ordination across the landscape and administrative boundaries. Also, the full potential complexity of local engagement becomes evident first at an aggregate level (Fig. 3). User groups interact and form social networks whose structures may both facilitate and constrain collective action towards ecosystem management and stewardship (Ernstson et al. 2008, 2010). The formation of co-management is channeled through the ability of civil society organization to build alliances between each other, and to government departments. It has been found that there are often more contacts between managers handling the same kind of area (e.g., cemeteries) than between neighboring green space managers, implying a neglect of plausible spatial ecological connections (Borgström et al. 2006; Ernstson et al. 2010). Actors able to connect over these boundaries, called brokers, are crucial as they greatly increase the opportunities for a diversity of actor groups to meet and exchange experiences. As historical (Walker 2007) and social movement research has indicated (Ansell 2003; Ernstson et al. 2008), urban green areas attracting a high diversity of interest and user groups seem to have higher chances of being protected and creating a social environment that nurture stewardship of ecosystem services because of increased potential for effective collective action and combination of knowledge and skills.

## Conclusion and Implications for Reconnecting Urban Areas to the Biosphere

The understanding of how urban ecosystems work, how they change, and what limits their performance, can add to the understanding of ecosystem change and governance in general in an ever more human-dominated world with implications for Earth Stewardship (Chapin et al. 2010). The high concentration of people, the diverse preferences that individuals, groups, business, and the state have for the city and the various demands for ecosystem services will cause continuous tension, which urban planning systems should be set up to handle. The importance and potential of urban planning also highlight the need for more research in the resource scarce cities of the Global south where the role played by planning is smaller.

Biodiversity and ecosystem services in urban landscapes are generated by complex interactions between ecological processes and human activities and organization. In an attempt to tackle this complexity, the scope of the research reported here includes social science in combination with systems ecology, ranging from local ecological knowledge as a strong connector between people and their environment to issues like learning, social memory, property rights, social movements, social justice, and cultural narratives. We have also highlighted the importance of including governance perspectives of legal protection, of actors in civil society, of brokers, and of environmental movements in the co-production of ecosystem services and biodiversity and the challenges of overcoming mismatches between the social and ecological systems both in space and time. This broad social–ecological approach on urban ecology has yielded a number of findings that should be of interest for this booming field of research:

1. Current urban planning strategies often fail to acknowledge ecological and social synergies. Distinct social–ecological dynamics in seemingly similar patches result in quite different and potentially complementing profiles of biodiversity and ecosystem services that might be lost if this stewardship is not understood or nurtured.
2. Small-scale land-use heterogeneity makes spatial organization especially important. The length and diversity of borders, biophysical as well as administrative, call for careful thinking to make sure adjacency effects are positive and that regulating ecosystem services reach across the urban landscapes.
3. Cities hold unexplored potential for new urban spatial designs that integrate ecosystem services in the built environment, for restoring degraded ecosystems and for strengthening ecosystem functions through complementary designs of land use and urban green structures.
4. Mismatches between social and ecological boundaries are prevalent. The meso-scale between local and regional is insufficiently addressed. Governance structures are needed that connect local experiential knowledge of ecosystem management with those of higher scale understanding. In such arrangements, the broker position in social networks should be identified and strengthened since it may be needed to link ecosystem stewards across scales, and on different sides of sectoral and administrative borders.
5. Different property rights lead to differences in practices, willingness to invest and learn about the system. Short-term tenure is more flexible while long term may lead to in-depth, situated knowledge and investment in restoration.
6. Participatory management approaches are critical for harnessing the diversity found within cities. These draw on diversity in the skill-bases that people and groups possess and also have the potential to provide more effective urban ecosystem management by taking into account multiple ways of knowing and evaluating urban land.

Cities could become laboratories where management strategies and governance structures for ecosystem stewardship are tested and evaluated. As most cities are not directly dependent on having all (especially provisioning) ecosystem services generated within-city boundaries, they are comparatively safe spaces for testing new governance structures and management practices within the domains where urban planning and design operate. For example, cities might be the best places to seek the answer to how diverse and contested interests in combination with limited space might be navigated to establish multifunctional land uses, an issue that will become increasingly important in many different social–ecological systems.

Cities arguably need to reduce their ecological footprint, but perhaps more importantly the character of the footprint need to change. A crucial step is to provide within-city opportunities for responsible stewardship to help reconnect citizens to the biosphere. In general, the promotion of “cognitive resilience building” for ecosystem stewardship in urban areas is central (Colding and Barthel 2013). It implies the perceptions, memory, and reasoning that people acquire from frequent interactions with local ecosystems, shaping peoples’ experiences, world views, and values toward local ecosystems and ultimately toward the biosphere. To achieve institutional changes, further studies are needed to explore the wider political processes that shape and promote how biophysical processes become articulated as of value, for example through the use of framings like ecosystem services. In a world where soon two-thirds of the population will live in cities both the individual and institutional level of analysis is of fundamental importance. Together with further research on the ecological underpinnings of ecosystem services, not least the cultural, future long-term urban social–ecological research must deepen our understanding of whether and how local stewardship and engagement in practical management of green infrastructures increase biodiversity and availability of ecosystem services in metropolitan landscapes, and if and how it actually stimulates a wider awareness and articulation of our global reliance on ecosystem services and results in an urban footprint both smaller and less detrimental to the resilience of the biosphere.

## Electronic supplementary material

Below is the link to the electronic supplementary material.

Supplementary material 1 (PDF 2125 kb)

## References

[CR1] Altieri MA, Companioni N, Cañizares K, Murphy C, Rosset P, Bourque M, Nicholls CI (1999). The greening of the “barrios”: Urban agriculture for food security in Cuba. Agriculture and Human Values.

[CR2] Andersson E, Bodin Ö (2009). Practical tool for landscape planning? An empirical investigation of network based models of habitat fragmentation. Ecography.

[CR3] Andersson E, Barthel S, Ahrné K (2007). Measuring social–ecological dynamics behind the generation of ecosystem services. Ecological Applications.

[CR4] Andrén H (1994). Effects of habitat fragmentation on birds and mammals in landscapes with different proportions of suitable habitat—A review. Oikos.

[CR5] Ansell CK, Diani M, McAdam D (2003). Community embeddedness and collaborative governance in the San Francisco Bay Area Environmental Movement. Social movements and networks—Relational approaches to collective action.

[CR7] Barthel S, Colding J, Elmqvist T, Folke C (2005). History and local management of a biodiversity-rich, urban, cultural landscape. Ecology and Society.

[CR8] Barthel S, Folke C, Colding J (2010). Social–ecological memory in gardening: Retaining the capacity for management of ecosystem services. Global Environmental Change.

[CR9] Berkes F, Folke C (1998). Linking social and ecological systems: Management practices and social mechanisms for building resilience.

[CR10] Blitzer EJ, Dormann CF, Holzschuh A, Kleind A-M, Rand TA, Tscharntke T (2012). Spillover of functionally important organisms between managed and natural habitats. Agriculture, Ecosystems and Environment.

[CR11] Borgström ST (2009). Patterns and challenges of urban nature conservation—A study of southern Sweden. Environment and Planning A.

[CR12] Borgström ST, Elmqvist T, Angelstam P, Alfsen-Norodom C (2006). Scale mismatches in management of urban landscapes. Ecology and Society.

[CR13] Borgström S, Cousins SAO, Lindborg R (2012). Outside the boundary—Land use changes in the surroundings of urban nature reserves. Applied Geography.

[CR14] Chapin FS, Carpenter SR, Kofinas GP, Folke C, Abel N, Clark WC, Olsson P, Stafford Smith DM (2010). Ecosystem stewardship: Sustainability strategies for a rapidly changing planet. Trends in Ecology & Evolution.

[CR15] Colding J (2007). “Ecological land-use complementation” for building resilience in urban ecosystems. Landscape and Urban Planning.

[CR16] Colding J, Barthel S (2013). The potential of “Urban Green Commons” in the resilience building of cities. Ecological Economics.

[CR17] Colding J, Folke C (2009). The role of golf courses in biodiversity conservation and ecosystem management. Ecosystems.

[CR18] Colding J, Lundberg J, Folke C (2006). Incorporating green-area user groups in urban ecosystem management. AMBIO.

[CR19] Elmqvist T, Folke C, Nyström M, Peterson G, Bengtsson J, Walker B, Norberg J (2003). Response diversity, ecosystem change, and resilience. Frontiers in Ecology and the Environment.

[CR20] Ernstson H, Sörlin S (2009). Weaving protective stories: Connective practices to articulate holistic values in the Stockholm National Urban Park. Environment and Planning A.

[CR21] Ernstson H, Sörlin S, Elmqvist T (2008). Social movements and ecosystem services—The role of social network structure in protecting and managing urban green areas in Stockholm. Ecology and Society.

[CR22] Ernstson H, Barthel S, Andersson E, Borgström ST (2010). Scale-crossing brokers and network governance of urban ecosystem services: The case of Stockholm, Sweden. Ecology and Society.

[CR23] Fahrig L, Baudry J, Brotons L, Burel FG, Crist TO, Fuller RJ, Sirami C, Siriwardena GM (2011). Functional landscape heterogeneity and animal biodiversity in agricultural landscapes. Ecology Letters.

[CR24] Foley JA, DeFries R, Asner GP, Barford C, Bonan G, Carpenter SR, Chapin FS, Coe MT (2005). Global consequences of land use. Science.

[CR25] Folke C, Jansson A, Larsson J, Costanza R (1997). Ecosystem appropriation by cities. AMBIO.

[CR26] Folke C, Colding J, Berkes F, Folke C, Berkes F, Colding J (2003). Building resilience and adaptive capacity in social–ecological systems. Navigating social–ecological systems: Building resilience for complexity and change.

[CR27] Folke C, Jansson Å, Rockström J, Olsson P, Carpenter SR, Chapin FS, Crépin A-S, Daily G (2011). Reconnecting to the biosphere. AMBIO.

[CR28] Goddard MA, Dougill AJ, Benton TG (2010). Scaling up from gardens: Biodiversity conservation in urban environments. Trends in Ecology & Evolution.

[CR29] Grimm NB, Faeth SH, Golubiewski NE, Redman CL, Wu J, Bai X, Briggs JM (2008). Global change and the ecology of cities. Science.

[CR30] Hanna S, Folke C, Mäler K-G (1996). Rights to nature: Ecological, economic, cultural, and political principles of institutions for the environment.

[CR31] Hobbs RJ, Arico S, Aronson J, Baron JS, Bridgewater P, Cramer VA, Epstein PR, Ewel JJ (2006). Novel ecosystems: Theoretical and management aspects of the new ecological world order. Global Ecology and Biogeography.

[CR32] Hope D, Gries C, Zhu WX, Fagan WF, Redman CL, Grimm NB, Nelson AL, Martin C (2003). Socioeconomics drive urban plant diversity. Proceedings of the National Academy of Sciences of the United States of America.

[CR33] Jansson Å, Nohrstedt P (2001). Carbon sinks and human freshwater dependence in Stockholm County. Ecological Economics.

[CR34] Jansson Å, Polasky S (2010). Quantifying biodiversity for building resilience for food security in urban areas: Getting down to business. Ecology and Society.

[CR35] Kinzig AP, Warren P, Martin C, Hope D, Katti M (2005). The effects of human socioeconomic status and cultural characteristics on urban patterns of biodiversity. Ecology and Society.

[CR36] Krasny M, Tidball K (2009). Community gardens as contexts for science, stewardship, and civic action learning. Cities and the Environment.

[CR37] Kremen C (2005). Managing ecosystem services: What do we need to know about their ecology?. Ecology Letters.

[CR38] Lee S, Webster C (2006). Enclosure of the urban commons. GeoJournal.

[CR39] Lundberg J, Andersson E, Cleary G, Elmqvist T (2008). Linkages beyond borders: Targeting spatial processes in fragmented urban landscapes. Landscape Ecology.

[CR40] Miller JR (2005). Biodiversity conservation and the extinction of experience. Trends in Ecology & Evolution.

[CR41] Nazarea DV (2006). Local knowledge and memory in biodiversity conservation. Annual Review of Anthropology.

[CR42] Ostrom E (1990). Governing the commons: The evolution of institutions for collective action.

[CR43] Poiani KA, Richter BD, Anderson MG, Richter HE (2000). Biodiversity conservation at multiple scales: Functional sites, landscapes, and networks. BioScience.

[CR44] Pyle RM (1978). The extinction of experience. Horticulture.

[CR45] Rees WE, Wackernagel M, Wackernagel M, Rees WE (1996). Urban ecological footprints: Why cities cannot be sustainable—And why they are a key to sustainability. Our ecological footprint, reducing human impact on the earth.

[CR46] Rockström J, Steffen W, Noone K, Persson Å, Chapin FS, Lambin EF, Lenton TM, Scheffer M (2009). A safe operating space for humanity. Nature.

[CR47] Runhaar HAC, Driessen PPJ, Soer L (2009). Scientific commons: Sustainable urban development and the challenge of policy integration: An assessment of planning tools for integrating spatial and environmental planning in the Netherlands. Environment and Planning B: Planning and Design.

[CR48] Seto KC, Fragkias M, Güneralp B, Reilly MK (2011). A meta-analysis of global urban land expansion. PLoS ONE.

[CR49] Turner WR, Nakamura T, Dinetti M (2004). Global urbanization and the separation of humans from nature. BioScience.

[CR50] Tzoulas K, Korpela K, Venn S, Yli-Pelkonen V, Kazmierczak A, Niemelä J, James P (2007). Promoting ecosystem and human health in urban areas using green infrastructure: A literature review. Landscape and Urban Planning.

[CR51] van Heezik YM, Dickinson KJM, Freeman C (2012). Closing the gap: Communicating to change, gardening practices in support of native biodiversity in urban private gardens. Ecology and Society.

[CR52] Walker C, Ntsebeza L, Hall R (2007). Redistributive land reform: For what and for whom?. The land question in South Africa: The challenge of transformation and redistribution.

[CR53] Wittemyer G, Elsen P, Bean WT, Coleman A, Burton O, Brashares JS (2008). Accelerated human population growth at protected areas edges. Science.

