# Supplementary material for: Reconnecting Cities to the Biosphere: Stewardship of Green Infrastructure and Urban Ecosystem Services
Source: Ambio. 2014 Apr 17;43(4):445–53. doi: 10.1007/s13280-014-0506-y (PMC3989518; doi:10.1007/s13280-014-0506-y)
Supplement: Supplementary file 1 — Supplementary material 1 (PDF 2125 kb) [file 13280_2014_506_MOESM1_ESM.pdf]

AMBIO

Electronic Supplementary Material

Reconnecting Cities to the Biosphere: Stewardship of Green Infrastructure and Urban Ecosystem Services

Erik Andersson, Stephan Barthel, Sara Borgström, Johan Colding, Thomas Elmqvist, Carl Folke, Åsa Gren

Appendix S1. Insights

- *Green areas:* The actual green space becomes three times larger when including domestic gardens, golf courses and allotment gardens than when only protected areas are considered, a common phenomenon in urban spatial planning (Colding et al. 2006). Sense of place and diversity of management practices enhance the potential for pollination, pest control and seed dispersal (Andersson et al. 2007). Many areas rich in biodiversity are managed for other purposes but provide essential ecosystem services (Barthel et al. 2005, Andersson et al. 2007, Colding and Folke 2009).
- *Mobile links:* Response diversity (Elmqvist et al. 2003) among pollinators is contingent on landscape structure and essential for sustaining crop production potential (Jansson and Polasky 2010). Birds, like the Jay breeding in coniferous forests are needed to sustain a biodiversity rich oak-dominated landscape high in recreational and cultural values (Lundberg et al. 2008).
- *Scales* and *boundaries:* Modelling supported by empirical investigation show that specialist bird species can be found in small urban habitat patches if supported by green landscape structure (Andersson and Bodin 2009). However, within the green areas different habitats are managed as if they were independent parcels. Local green area management is organized on a yearly basis and disconnected from large scale, long term green structure planning (Borgström et al. 2006, Ernstson et al. 2010).
- *Property rights:* At least 18 per cent of green space in Stockholm is managed by civil society (Colding et al. 2006) and in one of the largest green areas in the region engage 69 organizations, 25 of them as active stewards (Barthel et al. 2005, Barthel 2006). The steward groups are, however, only marginally connected to civil society protection networks (Ernstson et al. 2008). Length of lease holds leads to different kinds of learning and different habitat structure (Colding and Barthel 2013).

- *Social-ecological memory:* Green areas are endemic patches of an old cultural landscape and their values depend on continued management (Barthel et al. 2005, 2010). Memory carriers include imitation of practices and rituals, exchange of seeds, biological legacies, landscape morphology, stories, artefacts, tools and rules in use (Barthel et al. 2010). People with social-ecological memory are often ignored and marginalized the management of the cultural landscape in Stockholm (Ernstson et al. 2008, 2010).
- *Protection:* The area of protected land in Stockholm is planned to double 2003-2015. However, the linkages to the surrounding diverse urban landscape and potential civil society stewards are yet poorly developed (Borgström et al. 2006). The location of protected areas in urban landscapes is strongly influenced by other factors than ecological considerations, i.e. public support which in turn is dependent on social capital and economic status and class (Ernstson and Sörlin 2009).

Appendix S2. The social-ecological approach

The Stockholm school of urban ecological research views urban landscapes as integrated social-ecological systems (Berkes and Folke 1998) and emphasize the role of people, land use planning and management in the generation of urban ecosystem services (Fig. S1). Land use decisions, management practices, actors and institutional frameworks are understood as fundamental in stewardship of habitats and urban ecosystems for biodiversity and ecosystem services (Folke et al. 2011). The origin of the analytical approach derives from institutional analyses of long-term resource management in small-scale, local communities, where knowledge was developed from local cases that showed historic and successful management of ecosystem resilience and revealed management practices and social mechanisms with the capacity to cope with resource and ecosystem change (Berkes and Folke 1998, Berkes et al. 2003). The translation and application of this analytical framework to urban systems has been the most distinct trademark of the Stockholm school, with new insights generated on urban social-ecological systems and their dynamics.

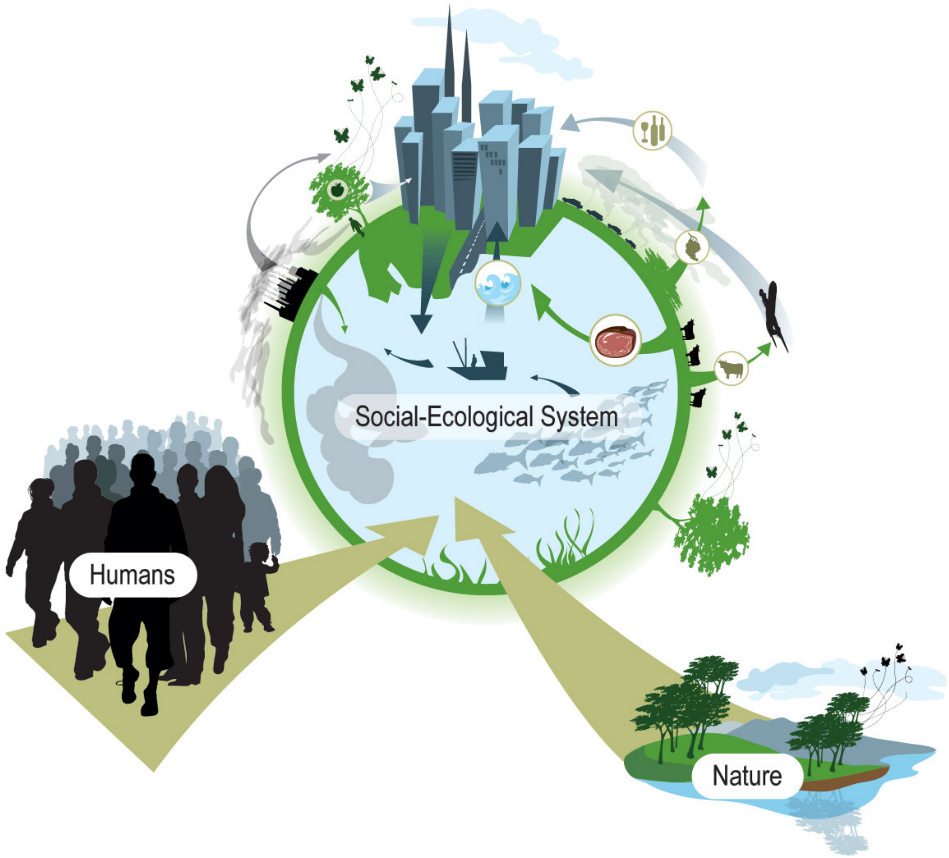

Fig. S1 The urban social-ecological approach to stewardship of green areas and ecosystem services. People and ecosystems are closely interconnected with strong feedbacks between social dynamics and ecosystem processes shaping development, in what we refer to as social-ecological systems (Berkes and Folke 1998). In an urban context recent social-ecological system research in cities connect issues like learning, experiences and memory, mismatches in space and time, user and property rights, narratives like sense of place, brokers and bridging organizations, co-production of ecosystem services, and role of social movements, formal institutions and planning for ecosystem stewardship.
